# Supplementary material for: Entanglement-Gradient Routing for Quantum Networks
Source: Sci Rep. 2017 Oct 27;7:14255. doi: 10.1038/s41598-017-14394-w (PMC5660278; doi:10.1038/s41598-017-14394-w)
Supplement: Supplementary file 1 — Supplemental Information [file 41598_2017_14394_MOESM1_ESM.pdf]

Entanglement-Gradient Routing for Quantum Networks

Laszlo Gyongyosi[1,2,3,*], Sandor Imre[3]

[1] School of Electronics and Computer Science
University of Southampton
Southampton SO17 1BJ, UK
[2] MTA-BME Information Systems Research Group
Hungarian Academy of Sciences
7 Nador st., Budapest, H-1051 Hungary
[3] Department of Networked Systems and Services
Budapest University of Technology and Economics
2 Magyar tudosok krt., Budapest, H-1117 Hungary

l.gyongyosi@soton.ac.uk

Supplemental Information

S.1 Source-Dependent Link Selection

The source-dependent link probability is derived as follows.

For the $A \to B$ scenario the results are analogous to (13). Therefore, utilizing $\mathcal{G}'^{y}_{z,B}$ in a current node y with neighbor node z, the $\Pr^{y}_{E_{\mathrm{L}_l}(y,z)}$ probability that from node y the entangled link $E_{\mathrm{L}_l}(y,z)$ is selected to reach destination B is evaluated as

$$\Pr^{y}_{E_{\mathrm{L}_l}(y,z)} = \frac{\left(\mathcal{G}'^{y}_{z,B} + \partial\right)^{\chi}}{\sum_k \left(\mathcal{G}'^{y}_{k,B} + \partial\right)^{\chi}} = \frac{\left(\left(\mathcal{G}^{y}_{z,B} e^{-\tau\left(\Delta B_F\left(E_{\mathrm{L}_l}(y,z)\right)\right)} + \lambda'_{E_{\mathrm{L}_l}(y,z)}\right) + \partial\right)^{\chi}}{\sum_k \left(\left(\mathcal{G}^{y}_{k,B} e^{-\tau\left(\Delta B_F\left(E_{\mathrm{L}_l}(y,k)\right)\right)}\right) + \partial\right)^{\chi}}, \tag{S.1}$$

where $\partial \geq 0$ is a threshold parameter, while $\chi \geq 0$ is a tuning parameter.

For the reverse direction $B \to A$, let $\Pr^{y}_{E_{\mathrm{L}_l}(x,y)}$ be the probability that from node y the entangled link $E_{\mathrm{L}_l}(x,y)$ is selected from y to reach source A.

The $\Pr^{y}_{E_{\mathrm{L}_l}(x,y)}$ entanglement gradient distribution is defined as

$$\Pr^{y}_{E_{\mathrm{L}_l}(x,y)} = \frac{\left(\mathcal{G}'^{y}_{A,x} + \partial\right)^{\chi}}{\sum_j \left(\mathcal{G}'^{y}_{A,j} + \partial\right)^{\chi}} = \frac{\left(\left(\mathcal{G}^{y}_{A,x} e^{-\tau\left(\Delta B_F\left(E_{\mathrm{L}_l}(x,y)\right)\right)} + \lambda'_{E_{\mathrm{L}_l}(x,y)}\right) + \partial\right)^{\chi}}{\sum_j \left(\mathcal{G}^{y}_{A,j} e^{-\tau\left(\Delta B_F\left(E_{\mathrm{L}_l}(j,y)\right)\right)} + \partial\right)^{\chi}}, \tag{S.2}$$

where x is the neighbor of y with entangled link $E_{\mathrm{L}_l}(x,y)$ with source node A.

S.1.1 Normalized Distribution

The normalized distribution $p^{y}_{E_{\mathrm{L}_l}(y,z)}$ that entangled link $E_{\mathrm{L}_l}(y,z)$ is selected from y to reach

From (S.1) and (S.2), the $p^{y}_{E_{\mathrm{L}_l}(y,z)}$ normalized probability distribution that entangled link $E_{\mathrm{L}_l}(y,z)$ is selected from y to reach destination B is as

$$p^{y}_{E_{\mathrm{L}_l}(y,z)} = \frac{\Pr^{y}_{E_{\mathrm{L}_l}(y,z)}\left(\Pr^{y}_{E_{\mathrm{L}_l}(x,y)}\right)^{-\xi}}{\sum_{k}\Pr^{y}_{E_{\mathrm{L}_l}(y,k)}\sum_{j}\left(\Pr^{y}_{E_{\mathrm{L}_l}(j,y)}\right)^{-\xi}}, \tag{S.3}$$

where $\xi \geq 0$ is a weight on the source entanglement gradient, while $\Pr^{y}_{E_{\mathrm{L}_l}(y,k)}$ is the probability that link $E_{\mathrm{L}_l}(y,k)$, where $k \in V - x$, will be selected at node y, evaluated as

$$\Pr^{y}_{E_{\mathrm{L}_l}(y,k)} = \frac{\left(\mathcal{G}'^{y}_{k,B} + \partial\right)^{\chi}}{\sum_{m \in V - x}\left(\mathcal{G}'^{y}_{m,B} + \partial\right)^{\chi}} = \frac{\left(\left(\mathcal{G}^{y}_{k,B}e^{-\tau\left(\Delta B_F\left(E_{\mathrm{L}_l}(y,k)\right)\right)} + \lambda'_{E_{\mathrm{L}_l}(y,k)}\right) + \partial\right)^{\chi}}{\sum_{m \in V - x}\left(\mathcal{G}^{y}_{m,B}e^{-\tau\left(\Delta B_F\left(E_{\mathrm{L}_l}(y,m)\right)\right)} + \partial\right)^{\chi}}, \tag{S.4}$$

while $\Pr^{y}_{E_{\mathrm{L}_l}(j,y)}$ is the probability that link $E_{\mathrm{L}_l}(j,y)$, where $j \in V - z$, will be selected at node y, expressed as

$$\Pr^{y}_{E_{\mathrm{L}_l}(j,y)} = \frac{\left(\mathcal{G}'^{y}_{A,x} + \partial\right)^{\chi}}{\sum_{g \in V - z}\left(\mathcal{G}'^{y}_{A,g} + \partial\right)^{\chi}} = \frac{\left(\left(\mathcal{G}^{y}_{A,x}e^{-\tau\left(\Delta B_F\left(E_{\mathrm{L}_l}(j,y)\right)\right)} + \lambda'_{E_{\mathrm{L}_l}(j,y)}\right) + \partial\right)^{\chi}}{\sum_{g \in V - z}\left(\mathcal{G}^{y}_{A,g}e^{-\tau\left(\Delta B_F\left(E_{\mathrm{L}_l}(g,y)\right)\right)} + \partial\right)^{\chi}}. \tag{S.5}$$

The model of the intermediate quantum network between A and B used for the derivation of (S.3) is illustrated in Fig. S.1. In the source-dependent network model, node A is also a source node for direct neighbor nodes x and j (e.g., exists a path between A and x, and between A and j), while node B is also a target node for direct neighbor nodes z and k (e.g., exists a path between z and B, and between k and B through an intermediate quantum network, respectively). The direct neighbors of y share an entangled link with the current node y.

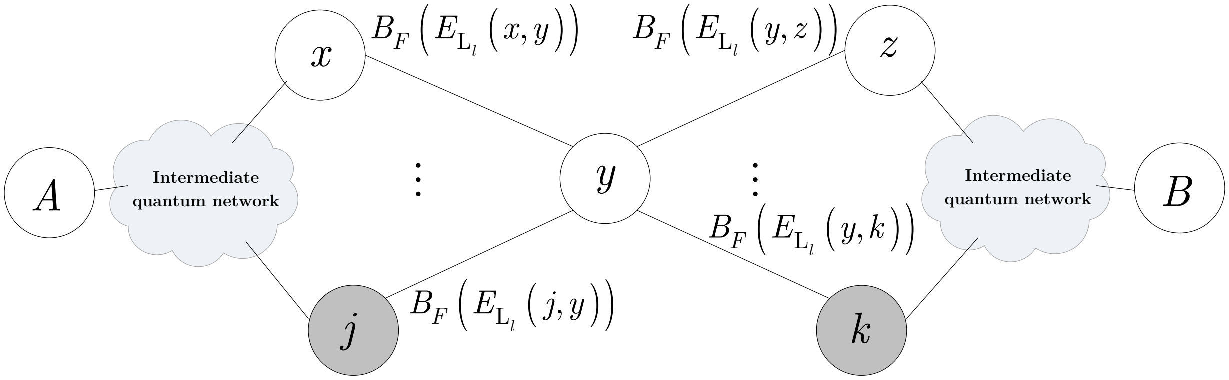

Figure S.1. Modeling source-dependent link selection through an intermediate network between quantum nodes A and B. The current node is y, with source node A and destination node B. The current direct neighbor of y with a path to source node A is x, while node z is the current direct neighbor of y with a path to target node B. Node j (gray) models a set of other neighbors of y with paths to source A, $j \in V - x$, while node k (gray) models a set of other neighbor of y with path to destination B, $k \in V - z$. Each direct neighbor has en entangled connection with y, denoted by $E_{\mathrm{L}_l}\left(x,y\right)$, $E_{\mathrm{L}_l}\left(y,z\right)$, and $E_{\mathrm{L}_l}\left(j,y\right)$, $E_{\mathrm{L}_l}\left(y,k\right)$. The entangled links are characterized by entanglement throughputs $B_F\left(E_{\mathrm{L}_l}\left(\cdot\right)\right)$, from which $\Delta B_F\left(E_{\mathrm{L}_l}\left(y,z\right)\right)$ is determined in node y to evaluate the entanglement gradient.

S.2 Notations

The notations of the manuscript are summarized in Table S.1.

Table S.1. Summary of notations.

Notation	Description
L1	Manhattan distance (L1 metric).
l	Level of entanglement.
F	Fidelity of entanglement.
N	Entangled quantum network, $N = \left(V, \mathcal{S}\right)$, where V is a

	set of nodes, $\mathcal{S}$ is a set of entangled links.
L_l	An l-level entangled link. For an L_l link, the hop-distance is 2^{l-1}.
$d\left(x,y\right)_{\mathrm{L}_l}$	Hop-distance of an l-level entangled link between nodes x and y, $d\left(A,B\right)_{\mathrm{L}_l} = 2^{l-1}$.
$E_{\mathrm{L}_l}\left(x,y\right)$	Entangled link $E_{\mathrm{L}_l}\left(x,y\right)$ between nodes x and y.
$\lambda_{E_{\mathrm{L}_l}\left(x,y\right)}$	Initial entanglement utility of link $E_{\mathrm{L}_l}\left(x,y\right)$.
$\lambda'_{E_{\mathrm{L}_l}\left(x,y\right)}$	Updated entanglement utility of link $E_{\mathrm{L}_l}\left(x,y\right)$.
$B_F\left(E_{\mathrm{L}_l}\left(x,y\right)\right)$	Entanglement throughput of a given L_l-level entangled link $E_{\mathrm{L}_l}\left(x,y\right)$ between nodes $\left(x,y\right)$.
$\mathcal{G}^{y}_{A,x}$	Initial entanglement gradient, from a source node A, on the neighbor node x at y, $\mathcal{G}^{y}_{A,x} \geq 0$.
	Updated entanglement gradient, from source node A, on the neighbor node x at y, $\mathcal{G}^{y}_{A,x} \geq 0$.
τ	Decay rate of entanglement gradient, $\tau \geq 0$.
$f\left(x\right)$	Probability distribution function.
$\Delta B_F\left(E_{\mathrm{L}_l}\left(x,y\right)\right)$	Deviation of entanglement throughput of a given link $E_{\mathrm{L}_l}\left(x,y\right)$ from an average, defined as $$\Delta B_F\left(E_{\mathrm{L}_l}\left(x,y\right)\right)=\left\lvert\frac{\sum_{h=1}^{n} B_F\left(E_{\mathrm{L}_l}\left(y,h\right)\right)}{n} - B_F\left(E_{\mathrm{L}_l}\left(x,y\right)\right)\right\rvert,$$ where n is the number of direct connections of node y, $\sum_{h=1}^{n} B_F\left(E_{\mathrm{L}_l}\left(y,h\right)\right)$ is the total entanglement throughput

	of all n direct links of node y, while $B_F\left(E_{\mathrm{L}_l}\left(x,y\right)\right)$ is the entanglement throughput of link $E_{\mathrm{L}_l}\left(x,y\right)$ between nodes y and x.
e^x	Exponential distribution function.
$X^y_{E_{\mathrm{L}_l}\left(x,y\right)}\left(t\right)$	Non-negative, non-stationary random process of entanglement utility $\lambda_{E_{\mathrm{L}_l}\left(x,y\right)}$.
$\mu^y_{E_{\mathrm{L}_l}\left(x,y\right)}\left(t\right)$	Mean of a non-negative, non-stationary random process $X^y_{E_{\mathrm{L}_l}\left(x,y\right)}\left(t\right)$.
$E\left[X^y_{E_{\mathrm{L}_l}\left(x,y\right)}\left(t\right)\right]$	Estimate of $X^y_{E_{\mathrm{L}_l}\left(x,y\right)}\left(t\right)$, defined as $$E\left[X^y_{E_{\mathrm{L}_l}\left(x,y\right)}\left(t\right)\right]=X^y_{E_{\mathrm{L}_l}\left(x,y\right)}\left(t\right)*\Omega_{\mathcal{G}^y_{A,x}}\left(t\right),$$ where $*$ is the convolution operator, while function $\Omega_{\mathcal{G}^y_{A,x}}\left(t\right)$ is defined as $$\Omega_{\mathcal{G}^y_{A,x}}\left(t\right)=e^{-\tau\left(\Delta B_F\left(E_{\mathrm{L}_l}\left(x,y\right)\right)\right)}U\left(t\right),$$ where $U\left(t\right)$ is the unit step function.
$\perp_{\mathcal{G}^y_{A,x}}\left(\Delta T\right)$	Correlation function, $\perp_{\mathcal{G}^y_{A,x}}\left(\Delta T\right)=e^{-\tau\left\|\Delta T\right\|}$, where ΔT is a time period.
$\Pr^y_{E_{\mathrm{L}_l}\left(y,z\right)}$	Link selection probability, probability that from node y the entangled link $E_{\mathrm{L}_l}\left(y,z\right)$ is selected to reach destination B.
∂	Threshold parameter, $\partial\geq 0$.
χ	Tuning parameter, $\chi\geq 0$.
$\mathcal{P}_i$	An i-th path between a source node A and target node B.
$\mathcal{G}^A_{\mathcal{P}_i}$	Initial path entanglement gradient of a given entangled

	path $\mathcal{P}_i$, $i=1,\ldots,m$ at source node A.
$\mathcal{G}_{\mathcal{P}_i}^{B}$	Initial path entanglement gradient of $\mathcal{P}_i$, $i=1,\ldots,m$ at destination node B.
κ_A	Observation rate, mean number of d-dimensional entangled states arrive in A.
κ_B	Observation rate, mean number of d-dimensional entangled states arrive in B.
κ_{AB}	Total observation rate, for a symmetrical arrival of the entangled states, $\kappa_A=\kappa_B=\kappa_{AB}/2$.
Z	Random variable, $Z=e^{-K\tau}$ where K is a random variable which models the interarrival time between the entangled states.
$\mu(K)$	Mean of random variable K.
$f_Z(x)$	Probability distribution function of Z, $$f_Z(x)=\frac{\kappa_{AB}}{\tau}x^{\left(\frac{\kappa_{AB}}{\tau}-1\right)},$$ where $0\leq x\leq 1$.
$\mu(Z)$	Mean of random variable $Z=e^{-K\tau}$, $$\mu(Z)=\frac{\kappa_{AB}}{\kappa_{AB}+\tau}=\gamma_{AB}.$$
$\mathcal{G}_{\mathcal{P}_i}^{\prime A}$	Updated path entanglement gradient the source node A for a given path $\mathcal{P}_i$, $i=1,\ldots,m$.
$\mathcal{G}_{\mathcal{P}_i}^{\prime B}$	Updated path entanglement gradient the source node B for a given path $\mathcal{P}_i$, $i=1,\ldots,m$.
$\mathcal{G}_{\mathcal{P}_j}^{\prime A}$	Updated path entanglement gradient the source node A for a given path $\mathcal{P}_j$, $j\neq i$.

$\mathcal{G}'^{B}_{\mathcal{P}_j}$	Updated path entanglement gradient the source node B for a given path $\mathcal{P}_j$, $j \neq i$.
$\mu^{A}_{\mathcal{P}_i}$	Average value of received entanglement gradient from path $\mathcal{P}_i$, $i = 1,\ldots,m$ at node A.
$\mu^{B}_{\mathcal{P}_i}$	Average value of received entanglement gradient from path $\mathcal{P}_i$, $i = 1,\ldots,m$ at node B.
$\mu^{A}_{\mathcal{P}_j}$	Average value of received entanglement gradient from path $\mathcal{P}_i$, $j \neq i$, at node A.
$\mu^{B}_{\mathcal{P}_j}$	Average value of received entanglement gradient from path $\mathcal{P}_i$, $j \neq i$, at node B.
$\mathcal{P}^{*}$	Optimal shortest path.
$\mathcal{G}'^{A}_{\mathcal{P}^{*}}$	Updated path entanglement gradient the source node A for optimal shortest path $\mathcal{P}^{*}$.
$\Pr^{A}_{\mathcal{P}_i}$	Probability that path $\mathcal{P}_i$, $i = 1,\ldots,m$ will be used by node A.
$\Pr^{B}_{\mathcal{P}_i}$	Probability that path $\mathcal{P}_i$, $i = 1,\ldots,m$ will be used by node B.
$\Pr^{A}_{\mathcal{P}_j}$	Probability that path $\mathcal{P}_j$, $j \neq i$, will be used by node A.
$\Pr^{B}_{\mathcal{P}_j}$	Probability that path $\mathcal{P}_j$, $j \neq i$, will be used by node B.
$\mathbb{E}\left(\mathcal{G}'^{A}_{\mathcal{P}_i}\right)$	Mean entanglement gradient of a particular path $\mathcal{P}_i$ at A, $i = 1,\ldots,m$.
$\mathbb{E}\left(\mathcal{G}'^{B}_{\mathcal{P}_i}\right)$	Mean entanglement gradient of a particular path $\mathcal{P}_i$ at B, $i = 1,\ldots,m$.
$\tau_{\mathbb{E}\left(\mathcal{G}'^{n}_{\mathcal{P}_i}\right)}$	Decay rate of mean path entanglement gradient $\mathbb{E}\left(\mathcal{G}'^{n}_{\mathcal{P}_i}\right)$.

$\partial_{\mathbb{E}\left(\mathcal{G}'^{n}_{\mathcal{P}_i}\right)}$	Threshold parameter to yield the $\tau_{\mathbb{E}\left(\mathcal{G}'^{n}_{\mathcal{P}_i}\right)}$ decay rate of mean path entanglement gradient.
$\tilde{\tau}_{\mathbb{E}\left(\mathcal{G}'^{n}_{\mathcal{P}_i}\right)}$	Optimal estimator of $\tau_{\mathcal{G}'^{n}_{\mathcal{P}_i}}$.
Y	Variable.
$B_F\left(\mathcal{P}_i\right)$	Entanglement throughput (measured in d-dimensional entangled states of a particular fidelity F per sec) of path $\mathcal{P}_i$
$\tilde{B}_F\left(\mathcal{P}_i\right)$	An expected $\tilde{B}_F\left(\mathcal{P}_i\right)$ entanglement throughput of a path $\mathcal{P}_i$.
$\varphi\left(\mathcal{P}_i\right)$	Deviation of a current $B_F\left(\mathcal{P}_i\right)$ entanglement throughput (measured in d-dimensional entangled states of a particular fidelity F per sec) of path $\mathcal{P}_i$ from an expected $\tilde{B}_F\left(\mathcal{P}_i\right)$ entanglement throughput of path $\mathcal{P}_i$, as $$\varphi\left(\mathcal{P}_i\right)=\left\lvert\tilde{B}_F\left(\mathcal{P}_i\right)-B_F\left(\mathcal{P}_i\right)\right\rvert.$$
$\Phi^{s,n}_{\mathcal{P}_i}$	Parameter for a given path $\mathcal{P}_i$, between a source node s and current node n, defined as $$\Phi^{s,n}_{\mathcal{P}_i}=\sum_{x=s}^{n}\alpha\sigma^{x}_{\mathcal{P}_i},$$ where α and $\sigma^{x}_{\mathcal{P}_i}$ are coefficients.
$\sigma^{x}_{\mathcal{P}_i}$	Coefficient used by $\Phi^{s,n}_{\mathcal{P}_i}$, $$\sigma^{x}_{\mathcal{P}_i}=\log\left(\frac{\mathcal{G}'^{x+1\in\mathcal{P}_i}_{\mathcal{P}_i}}{\mathcal{G}'^{x\in\mathcal{P}_i}_{\mathcal{P}_i}}\right),$$ where $\mathcal{G}'^{x\in\mathcal{P}_i}_{\mathcal{P}_k}$ is the entanglement gradient of node $x\in\mathcal{P}_i$,

	while $\mathcal{G}'^{x+1\in\mathcal{P}_i}_{\mathcal{P}_k}$ is the entanglement gradient at node $x+1\in\mathcal{P}_i$.
α	Coefficient used by $\Phi^{s,n}_{\mathcal{P}_i}$, defined as $$\alpha=\begin{cases}1, & if\ \left\|\sigma^x_{\mathcal{P}_i}\right\|>\vartheta\\ 0, & if\ \left\|\sigma^x_{\mathcal{P}_i}\right\|\leq\vartheta\end{cases},$$ where ϑ is a threshold.
ϑ	Threshold parameter.
$\mu^n\left(\Phi^{s,n}_{\mathcal{P}}\right)$	Mean for the m paths $\mathcal{P}_1,\ldots,\mathcal{P}_m$ between a source node s and a current node n, as $$\mu^n\left(\Phi^{s,n}_{\mathcal{P}}\right)=\frac{\sum_{i=1}^{m}\Phi^{s,n}_{\mathcal{P}_i}}{m}.$$
$\psi\left(n,z\right)$	A distance function $\psi\left(n,z\right)$ between n and z.
$\mathbb{E}\left(\mathcal{G}'^n_{\mathcal{P}_i}\right)$	Mean entanglement gradients at node $n\in\mathcal{P}_i$.
$\mathbb{E}\left(\mathcal{G}'^z_{\mathcal{P}_i}\right)$	Mean entanglement gradients at node $z\in\mathcal{P}_i$.
$\theta^z_{E_{\mathrm{L}_l}\left(n,z\right)}$	Inverse link entanglement gradient, $$\theta^z_{E_{\mathrm{L}_l}\left(n,z\right)}=\frac{1}{\mathcal{G}'^z_{A,n}}=\frac{1}{\mathcal{G}^z_{A,n}e^{-\tau\left(\Delta B_F\left(E_{\mathrm{L}_l}\left(n,z\right)\right)\right)}+\lambda'_{E_{\mathrm{L}_l}\left(n,z\right)}},$$ where $\lambda'_{E_{\mathrm{L}_l}\left(n,z\right)}$ is the updated entanglement utility, as $$\lambda'_{E_{\mathrm{L}_l}\left(n,z\right)}=\frac{\lambda_{E_{\mathrm{L}_l}\left(n,z\right)}}{1+B_F\left(E_{\mathrm{L}_l}\left(n,z\right)\right)\lambda_{E_{\mathrm{L}_l}\left(n,z\right)}}.$$
t	Number of threads.
$\mathcal{T}_i$	An i-th thread, $\mathcal{T}_1,\ldots,\mathcal{T}_t$.
$\ell_{\mathcal{T}}$	Thread-threshold, limits maximal number of nodes visited

	by a given thread to at most $\ell_{\mathcal{T}}$.
$p_{\mathcal{T}_i}(n,z)$	Link selection probability for an i-th thread $\mathcal{T}_i$.
$\mathrm{S}_{\mathcal{T}_i}$	A set of nodes already visited by the i-th thread $\mathcal{T}_i$.
$\mathrm{Pr}_{\mathcal{T}_i}(n,z)$	Probability function for an i-th thread $\mathcal{T}_i$.
C_1, C_2	Weighting parameters to balance the relevance between inverse entanglement gradient function $\theta(\cdot)$ and distance function $\psi(\cdot)$ in $\mathrm{Pr}_{\mathcal{T}_i}(n,z)$.
ν_n, $\varsigma(\gamma_n)$, $\rho(\nu_n)$	Additional parameters.
γ_n	Parameter for a node n, defined as $$\gamma_n = \frac{\kappa_n}{\kappa_n + \tau_n} = \left(1 + \frac{\tau_n}{\kappa_n}\right)^{-1},$$ where κ_n is the observation rate in node n, mean number of d-dimensional entangled states arrive in n, τ_n decay rate of entanglement gradient in node n.
Π	Tuning parameter (a fraction of peak value), $0 \leq \Pi \leq 1$.
κ_n^*	Cutoff observation rate (critical value of received d-dimensional entangled states per sec) defined at a given observation rate κ_n, controllable by τ_n.
$\mathcal{R}$	Ideal recovery operation with an optimal quantum error correction.
$\tilde{\Psi}$	Shared Bell pair between the final stations.
ρ_f	Input density matrix of ideal recovery operation $\mathcal{R}$.
P_{err}	Per-node error probability P_{err}, includes the effective logical error probability Q and other residual errors ε_{res} in

	the node.
$\mathcal{M}(A,B)$	Correlation measurement between the final stations A and B, yields entanglement fidelity as $\mathcal{M}(A,B) \approx \sqrt{F}$.
